# Genetic Basis of Primary Angle Closure Glaucoma: The Role of Collagens and Extracellular Matrix

**DOI:** 10.18502/jovr.v15i1.5930

**Published:** 2020-02-02

**Authors:** Elahe Elahi

**Affiliations:** ^1^ Department of Biotechnology, College of Science, University of Tehran, Tehran, Iran

Glaucoma is a heterogeneous group of optic neuropathies characterized by a specific pattern of optic nerve degeneration and visual field loss that is usually accompanied by increased intraocular pressure (IOP).^[1]^ It is a major cause of irreversible blindness worldwide.^[2]^ Primary glaucoma is classified into three major forms based on the anatomy of the anterior chamber drainage angle of the eye and the age of onset: primary congenital glaucoma (PCG), primary open angle glaucoma (POAG), and primary angle closure glaucoma (PACG).^[1]^ In glaucoma patients with increased IOP, the increase is thought to be mainly due to impaired drainage of aqueous humor from the anterior chamber.^[3]^

The etiology of all forms of glaucoma includes a genetic component as evidenced by variable prevalence in different ethnic groups, observations on familial clustering, and results of pedigree and sib-pair studies. For PCG, which is usually a monogenetic Mendelian disease, three causative genes have been identified.^[4,5,6,7]^ POAG and PACG are generally considered complex multifactorial disorders.^[8]^ Several POAG-causing genes have been identified, but mutations in these account for disease in less than 10% of patients.^[9,10,11,12,13]^

Compared to the other forms of glaucoma, there is much less definitive genetic data pertaining to PACG. This likely reflects the contribution of multiple genetic and perhaps environmental factors that affect various anatomical and functional features associated with PACG. PACG is an important public health entity. It is estimated that 15.7 million individuals in the world are affected with PACG. It is projected that 21 million will be affected by 2020, and that PACG by that time will cause bilateral blindness in 5.3 million people.^[14,15,16]^ Most PACG patients are from Asia, particularly China, Mongolia, Singapore, and India.

Ultimately, the defining feature of PACG in individuals with glaucomatous optic nerve damage is iridocorneal angle closure. Recently, *COL18A1* which encodes collagen type XVIII was identified as a gene that affects angle closure in patients of three unrelated families.^[17]^ The inheritance pattern of angle closure causing mutations in *COL18A1* was autosomal dominant. It appears that mutations in this gene may cause angle closure in the fourth decade of life or later. Furthermore, *COL18A1* mutations are not expected to be a common cause of angle closure-related phenotypes. The significance of having identified *COL18A1* as a potential PACG-causing gene lies in emphasis on the importance of collagens and the extracellular matrix in glaucoma pathology.

In pursuit of identifying genes that contribute to PACG disease, association studies and candidate gene studies have been performed and the results of these have implicated possible roles for several genes.^[18,19,20,21,22,23,24,25]^ However, these findings are not generally considered definitive, and putative roles of genes suggested by some studies were not confirmed in independent studies. Factors known to be associated with PACG include hyperoptic refractive error, shallow anterior chamber, thick crystalline lens, short axial length, small corneal diameter, and narrow iridocorneal angle.^[26,27,28]^ Clearly, some of these also associate with each other. In an article published in this issue of the *Journal of Ophthalmic and Vision Research*, the authors relied on transcriptome data pertaining to eye anterior segment tissues to select five single nucleotide polymorphisms (SNPs) whose genotypes may be associated with PACG among patients of Northeast Iran.^[29,30,31]^ Interestingly, one of the SNPs for which an association with PACG was reported is positioned in a gene (*FERMT2*) that encodes a component of the ECM. Results of an association study that included tens of thousands of patients and controls had also implicated the same gene with respect to PACG.^[20]^

There exists accumulating evidence that implicates various collagens specifically and the extracellular matrix more generally in the pathogenesis of glaucoma.^[7,32,33,34,35,36]^ Eight loci were identified in one or both of two recent large genome-wide association (GWA) studies on PACG patients, and *COL11A1 *that encodes one of the alpha chains of type XI collagen was one of the genes identified in both of the studies.^[18,20]^ An SNP in *COL1A1* was associated with increased risk of myopia in Japanese and Chinese individuals.^[37,38]^ A GWA study of PACG in a dog breed identified *COL1A2* as a susceptibility locus.^[39]^ And of course, *COL18A1* was identified as a gene that affects iridocorneal angle closure in humans.^[17]^ In addition to collagens, genes with roles in the extracellular matrix and also associated with glaucoma include *MMP-9*, *MTHFR*, *LTBP2*, *CYP1B1*, and *SPARC*. It has been suggested that inter-individual differences in tolerance to IOP as reflected in glaucoma diagnosis with normal tension in some and ocular hypertension without glaucoma in others reflect variations in biomechanical properties of the extracellular matrix of relevant ocular tissues.^[32]^
